# Prevention of Cholera in New York

**Published:** 1866-10

**Authors:** 


*Prevention of Cholera in New York.*—We quote the following interesting statements from Prof. A. Clark's Lectures on Cholera, recently published in the *Medical Record:*

One discovery, growing out of theory to be sure, but itself worth more than all theories, has been strikingly confirmed in numerous instances the present year—the power of certain chemicals, called disinfectants, aided by the removal of filth, to stop the progress of cholera in limited localities, as a house or portion of a street, and by inference in all houses and streets. It is difficult, perhaps impossible, to trace relations between individual cases in a large city; but once more we have the fact that cholera did not appear in New York till after infected vessels had arrived in our harbor. If we can trust the newspaper reports, the disease broke out among the troops on Hart's Island, Governor's Island, and on a transport carrying soldiers to Tybee Island, only after sending to these places, and by this ship, recruits newly arrived in emigrant vessels; but wherever it has appeared within the jurisdiction of the Board of Health, out of public institutions, it has been met with a promptness and vigor not unexpected from such men as constitute that Board and deserving the highest praise. For three and a half months there have been scattered cases, and a few times small groups of cases occurring here; but I believe I announce the literal truth when I say that it has been promptly driven out of every house it has entered. The officers of the Board are ready at all hours, with their disinfectants distributed in wagons, horses harnessed, and medical officers in waiting, to drive to and disinfect any and every house where the disease appears. It has been thus systematically pursued from its very first occurrence to this moment. It has not been finally hunted down, and probably will not be till near winter, because the disease is rapidly increasing in the commercial towns of Europe and also in the interior districts of Germany, from which emigrants are daily arriving. In the month of May it is reported that 40,000 emigrants were landed in New York. Notwithstanding the vigilance of the quarantine officers, some of these will carry cholera with them, either by protracted incubation, or unreported diarrhœa, or in their unpurified baggage, and it will break out anew among us. The present indications are that deaths from cholera on Manhattan Island during the year will not exceed 500, while the deaths from ordinary causes will be as heretofore 22,000 or more. This low mortality is due to the unwearied and successful efforts of the Board of Health to expel it from every new lodgment it makes, to purify the city, and admonish citizens of their personal and domestic obligations. Up to August 17th, I am officially informed, excluding the islands in the East River, the deaths from cholera in New York have been only 247.

